# Enhanced Solubility, Permeability and Anticancer Activity of Vorinostat Using Tailored Mesoporous Silica Nanoparticles

**DOI:** 10.3390/pharmaceutics10040283

**Published:** 2018-12-17

**Authors:** Anand Kumar Meka, Laura J. Jenkins, Mercedes Dàvalos-Salas, Naisarg Pujara, Kuan Yau Wong, Tushar Kumeria, John M. Mariadason, Amirali Popat

**Affiliations:** 1 School of Pharmacy, The University of Queensland, Brisbane, QLD 4102, Australia; a.meka@uq.edu.au (A.K.M.); n.pujara@uq.edu.au (N.P.); t.kumeria@uq.edu.au (T.K.); 2 Olivia Newton-John Cancer Research Institute, La Trobe University School of Cancer Medicine, Melbourne, VIC 3084, Australia; Laura.Jenkins@onjcri.org.au (L.J.J.); mercedes.davalos@onjcri.org.au (M.D.-S.); 3 Mater Research Institute—The University of Queensland, Translational Research Institute, Woolloongabba, QLD 4102, Australia; kuanyau.wong@mater.uq.edu.au

**Keywords:** vorinostat, cancer, drug delivery, porous silica, porous materials, drug delivery, particle cell interaction, BCS classification

## Abstract

Suberoylanilide hydroxamic acid (SAHA) or vorinostat (VOR) is a potent inhibitor of class I histone deacetylases (HDACs) that is approved for the treatment of cutaneous T-cell lymphoma. However, it has the intrinsic limitations of low water solubility and low permeability which reduces its clinical potential especially when given orally. Packaging of drugs within ordered mesoporous silica nanoparticles (MSNs) is an emerging strategy for increasing drug solubility and permeability of BCS (Biopharmaceutical Classification System) class II and IV drugs. In this study, we encapsulated vorinostat within MSNs modified with different functional groups, and assessed its solubility, permeability and anti-cancer efficacy in vitro. Compared to free drug, the solubility of vorinostat was enhanced 2.6-fold upon encapsulation in pristine MSNs (MCM-41-VOR). Solubility was further enhanced when MSNs were modified with silanes having amino (3.9 fold) or phosphonate (4.3 fold) terminal functional groups. Moreover, permeability of vorinostat into Caco-2 human colon cancer cells was significantly enhanced for MSN-based formulations, particularly MSNs modified with amino functional group (MCM-41-NH_2_-VOR) where it was enhanced ~4 fold. Compared to free drug, vorinostat encapsulated within amino-modified MSNs robustly induced histone hyperacetylation and expression of established histone deacetylase inhibitor (HDACi)-target genes, and induced extensive apoptosis in HCT116 colon cancer cells. Similar effects were observed on apoptosis induction in HH cutaneous T-cell lymphoma cells. Thus, encapsulation of the BCS class IV molecule vorinostat within MSNs represents an effective strategy for improving its solubility, permeability and anti-tumour activity.

## 1. Introduction:

The efficient oral delivery of hydrophobic molecules to target tissues represents a major clinical challenge [1,2]. Over the past decade, there has been significant interest in the use of porous nanomaterials for a range of biomedical applications [3,4,5]. In particular, there has been extensive interest in the use of porous nanoparticles for drug delivery to tumours based on their potential to preferentially accumulate within tumours as a result of their abnormal vasculature and lack of lymphatics (Enhanced Permeation and Retention (EPR) effect) [6,7,8,9]. In addition, there has also been growing interest in the use of porous nanomaterials for improving the oral delivery of small hydrophobic molecules and macromolecules [10,11]. In this regard, mesoporous silica-based nanoparticles (MSNs) have particularly strong potential as drug delivery vehicles due to their high and tuneable surface area (>1000 m^2^/g), good biocompatibility, uniform particle size, tuneable pore sizes, large pore volumes, thermal stability and ease of functionalisation [6,7,8,12,13,14]. In the context of oral drug delivery, MSNs with pore sizes in the nanometre range improve the solubility of hydrophobic drugs by maintaining crystalline drugs in an amorphous state while loading, without altering their lattice energy [15,16]. Additionally, confined nanopores prevent further nucleation, slowing or stopping recrystallisation of adsorbed substrates and keeping them in a stable low energy amorphous state. The pore size of MSNs can also be tuned on a nanoscale to regulate the confinement of drug molecules, which reduces the size of the drug particles and increases drug solubility [17,18]. Furthermore, the large surface area of MSNs provides rapid adsorption of cargo including encapsulation of genetic materials [19,20]. Finally, the surface functionalisation of MSNs can provide stimuli responsive drug release based on external or internal stimuli for more targeted drug delivery [21,22,23]. 

Importantly, the solubility and oral bioavailability of several BCS class II drugs (drugs with low solubility and high permeability) has been shown to be improved by encapsulation within mesoporous materials with different particle shape, size, surface functionalisation and pore sizes [12,14,24,25,26,27,28,29,30]. Comparatively, only a few studies have tested the potential of MSNs for improving the physicochemical and biological activity of BCS class IV molecules with both poor solubility and permeability [31,32]. Specifically, two studies reported improvement in the in vitro dissolution profile of furosemide; however, the effect on saturated solubility or permeability was not reported [31,32]. In a separate study, furosemide was dissolved in lipids and mixed with non-porous silica aerosil^®^ to form microparticles which improved its ex vivo permeation [33]. However, these types of formulations suffer from low encapsulation efficiency requiring high amounts of multiple lipids/surfactants, while undesirable precipitation of crystalline drug due to partitioning during digestion or dispersion is also common [1].

In this study, we sought to determine the effect of encapsulation of the BCS class IV molecule vorinostat within functionalised MSNs on its solubility, permeability and anti-cancer activity. Vorinostat is approved for the treatment of cutaneous T-cell lymphoma, and is a member of the hydoxamic acid class of histone deacetylase inhibitors (HDACi), which inhibit the class I and II HDAC family of transcriptional co-repressors [34,35]. Consequently, HDACi induce profound changes on gene expression in tumour cells, which results in anti-tumour activity through a variety of mechanisms, particularly apoptosis induction. Due to its poor solubility and permeability, vorinostat has poor oral bioavailability which limits its clinical application [36,37]. The approved marketed oral formulation ZOLINZA^®^ contains 100 mg of vorinostat and has to be taken up to 4 times a day due to poor oral bioavailability. The parenteral formulations of this drug also suffer from poor half-life in the blood (40 mins) after intravenous administration [38]. We hypothesised that use of nanostructured materials such as mesoporous silica as a delivery vehicle may significantly improve the physicochemical properties of BCS class IV molecules such as vorinostat.

Here, we demonstrate that the solubility and permeability of vorinostat can be significantly improved by encapsulation within functionalised MCM-41 type mesoporous silica nanoparticles, with high loading capacity (~15–20%) achieved using a rotatory evaporation method. The improvement in solubility also resulted in improved permeability, especially when encapsulated within positively charged silica particles. We also demonstrate that the ability of vorinostat to inhibit HDAC activity, alter gene expression and induce apoptosis in colorectal cancer and cutaneous T-cell lymphoma cells is markedly enhanced compared to free drug, and are similar to the effects induced by vorinostat solubilised in DMSO. To the best of our knowledge this is the first study that demonstrates efficient encapsulation of vorinostat within highly ordered MSNs with marked improvement in physicochemical and anti-cancer activity.

## 2. Experimental Section

### 2.1. Materials

Cetyltrimethylammonium bromide (CTAB, 99%), tetraethyl orthosilicate (TEOS, reagent grade 98%), (3-Aminopropyl) triethoxysilane (APTES, 99%), 3-(trihydroxy silyl) propyl methyl phosphonate (THMP, 90%), dimethyl sulfoxide (DMSO), Toluene (ACS), Acetone (analytical grade), hydrochloric acid (32%) Acetonitrile (HPLC grade), Trifluoro acetic acid were purchased from MERCK, Frenchs Forest, Australia. Vorinostat was purchased from Sapphire Bioscience Pty. Ltd., Redfern, Australia. All aqueous solutions were prepared using DI water (18 MΩ) from Millipore Milli-Q system.

### 2.2. Synthesis of MCM-41

MCM-41 particles were prepared using a previously published protocol with slight modification [30]. Briefly, CTAB (1 g) was dissolved in 480 mL of deionised water to which 3.5 mL of NaOH (2 M) was added, and the temperature raised to 80 °C. TEOS (6.7 mL) was slowly added to this mixture and stirred for 2 h at 80 °C. The sample suspension was then vacuum filtered and washed with deionised water. The filtrate was dried overnight at room temperature followed by calcination at 550 °C for 5 h in a muffle furnace at a temperature ramp rate of 5 °C/min for up-ramp and 10 °C/min for down-ramp. 

### 2.3. Surface Functionalisation of MCM-41

Amino and phosphonate modification of MSNs was performed as previously described with slight changes [39,40]. For amino functionalisation, 400 mg of MCM-41 particles were dispersed in 60 mL toluene and stirred at 50 °C for 30 mins. APTES (400 µL) was then added and refluxed overnight at 110 °C. MCM-41-NH_2_ particles were obtained by centrifugation at 15,000 rpm for 10 min, washing three times in ethanol, and drying overnight at room temperature. For phosphonate functionalisation, 400 mg MCM-41 particles were dispersed in 65 mL of deionised water containing THMP (400 µL). The pH was adjusted to ~pH 5–6 to avoid hydroxylation and condensation of silanol groups on MCM-41 during the functionalisation. The mixture was refluxed overnight at 100 °C, and functionalised particles collected by centrifugation at 15,000 rpm for 10 min, washing with deionised water and ethanol three times, and drying at room temperature.

### 2.4. Characterisation of MCM-41

Transmission electron microscopy (TEM) images of MCM-41 nanoparticles were obtained using a Hitachi 7700 microscope operated at 100 kV. Pore dimension, volume and specific surface were obtained using nitrogen absorption (N_2_-BET) (Tristar, Micromeritics-II, Norcross, GA, USA). The pore size distribution was measured from the adsorption branch of the isotherm using the BJH model, and the particle size and surface charge measured using dynamic light scattering (DLS) and zeta potential measurements (Malvern, Nano-ZS, ATA Scientific, Taren Point, Australia). Thermogravimetric analysis (TGA) (Mettler Toledo, TGA/DSC 2, Columbus, OH, USA) was performed with a heating rate of 5 °C/min in air flow. X-ray diffractograms and wide angle XRD were recorded using a Bruker X-ray diffractometer with Cu radiation (λ = 1.54 °A). Vorinostat concentrations were determined using the HPLC method based on existing literature with slight modification [37]. Vorinostat was eluted isocratically with mobile phase (Acetonitrile/Water/Acetic acid at ratio of 40/60/0.1%) at flow rate of 1 mL/min, 10 ul injection volume and monitored at using UV detector at 260 nm (R^2^ = 0.9986). Chromatograms were recorded and integrated with lab solutions software.

### 2.5. Loading of Vorinostat into MSNs and Functionalised MSN Particles

Vorinostat was loaded into the pristine and functionalised MCM-41 particles through a simple incubation followed by rotary evaporation as per our previous reports [28,30]. To obtain 20% mass loading, 20 mg of vorinostat was dissolved in 10 mL of acetone, followed by addition of 80 mg of either pristine or functionalised MCM-41 nanoparticles. The mixture was stirred overnight in the dark at room temperature, and the solvent evaporated using a rotary evaporator at 40 °C. The amount of vorinostat loading was determined using TGA by computing the weight loss as a function of temperature and comparison to respective blank particles.

### 2.6. Vorinostat Solubility from MCM-41 and Functionalised Particles

To determine the solubility of vorinostat after loading into MSNs, 0.5 mg of drug equivalent particles (i.e., 2.5 mg of ~20 wt. % vorinostat loaded MSNs) were dispersed in 500 µL of deionised water. Samples were maintained at 37 °C and stirred constantly. After 48 h, samples were centrifuged at 148,00 rpm for 10 min at room temperature. To completely remove the MSN particles, 400 µL of the supernatant was centrifuged again under the same conditions, following which the supernatant was diluted appropriately and analysed using a HPLC method reported earlier with slight modification [37]. 

### 2.7. Caco-2 Permeability Experiments

The permeability of vorinostat in vitro was determined using the Caco-2 cell line monolayer assay. Caco-2 cells (1 × 10^5^ cells/well) were seeded in 12-well cell culture inserts (1 µm pore diameter, 0.9 cm^2^ area) and cultured in DMEM supplemented with 1% PEST and 1% Glutamine for 10–14 days. Transepithelial electrical resistance (TEER) was determined using an EVOM volt-ohmmeter (Coherent Scientific, Hilton, Australia). Only Caco-2 cell monolayers with initial TEER values ~500–600 Ω cm^2^ were used. For permeability studies, 10, 25, and 50 µg/mL of vorinostat alone (pre-dissolved in 1% DMSO) or equivalent concentration of vorinostat loaded in MSNs were suspended in Hanks Balanced Salt Solution (HBSS), and added to the apical compartment. Blank medium (HBSS) and nanoparticles with no drug at a concentration equivalent to that used for vorinostat (50 µg/mL) were used as controls. The basolateral compartment (receiver compartment) was filled with 1.2 mL of HBSS + 10 mM hydroxyethyl piperzineethanesulfonic acid (HEPES). After 4 h incubation, samples were collected from the basolateral compartment and the concentration of vorinstat determined by HPLC. To test the effect of nanoparticles (both loaded and unloaded) and nanoparticles loaded with vorinostat on TEER, particles and vorinostat were removed after the 4 h incubation and replaced with cell culture media, and TEER was measured over the following 24 h.

### 2.8. Cell Culture

The colorectal cancer cell line HCT116 and the cutaneous T cell lymphoma cell line HH were obtained from the American Type Culture Collection (ATCC, Manassas, VA, USA). Cells were maintained at 37 °C and 5% CO_2_ in DMEM/F-12 and RPMI-1640, respectively and supplemented with 10% foetal calf serum (FCS) and 2 mmol L-glutamine as previously described [41]. 

### 2.9. Histone Extraction and Western Blotting

HCT116 cells were treated with increasing concentrations of vorinostat pre-dissolved in DMEM, DMSO, or loaded within MCM-41-NH_2_ particles, for 1 h, and cell pellets collected by scraping in cold PBS. For histone extraction, cells pellets were lysed in 100 µL cold histone lysis buffer (10 mM HEPES pH 7.9, 1.5 mM MgCl_2_, 10 mM KCl and 0.5 mM DTT) supplemented with protease inhibitor (complete, Roche, Basel, Switzerland) and 4 µL of 5 M sulphuric acid, for 1 h with intermittent vortexing. Samples were centrifuged at maximum speed for 10 min at 4 °C, the supernatant harvested and mixed with acetone at a ratio of 9 (acetone): 1 (lysate), and incubated overnight at −20 °C. Samples were then centrifuged at maximum speed for 10 min at 4 °C, pellets washed in 70% ethanol, air dried and resuspended in H_2_O. 15 µg of protein was resolved on NuPAGE Novex 4–12% Bis-Tris gels (Invitrogen, Carlsbad, CA, USA) and transferred onto a PVDF membrane (Invitrogen, Carlsbad, CA, USA). Membranes were blocked using Odyssey PBS blocking buffer (LI-COR, Lincoln, NE, USA) for 1 h and incubated with rabbit anti-Ac. Histone H3 (06-599, Merck Millipore, Burlington, MA, USA, 1:10,000) or goat anti-Histone H3 (sc8654, Santa Cruz Biotechnology, Dallas, TX, USA, 1:5000) for 1 h at room temperature, and subsequently with the corresponding species of fluorescent IRDye secondary antibody (LI-COR, Lincoln, NE, USA). Signal was visualised using the Odyssey Classic Infrared Imaging System and Odyssey software (LI-COR, Lincoln, NE, USA). 

### 2.10. Quantitative RT-PCR

Total RNA was purified using the ReliaPrep^TM^ RNA miniprep system (Promega, Madison, WI, USA) and reverse transcribed using random hexamers from the High-Capacity cDNA Reverse Transcription Kit (Applied Biosystems, Foster City, CA, USA), according to the manufacturer’s instructions. Quantitative RT-PCR was performed using Power SYBR Green PCR Master Mix (Applied Biosystems, Foster City, CA, USA) on the ViiA 7 Real Time PCR system (Applied Biosystems, Foster City, CA, USA) according to manufacturer’s instructions. cDNA (5 ng) was amplified with 75 nM forward and reverse primers in a 5 µL total reaction. Primers used were as follows: *ATF3* F: GTGCCGAAACAAGAAGAAGG; *ATF3* R: CGAGAGGAAGATGGGAGATG; *JUN* F: TGACTGCAAAGATGGAAACG; *JUN* R: TGAGGAGGTCCGAGTTCTTG; *GAPDH* F: ATGGAAATCCCATCACCATCTT; *GAPDH* R: CGCCCCACTTGATTTTGG.

### 2.11. Apoptosis Assays

HCT116 and HH cells were seeded in triplicate in 24-well plates at a density of 50,000 cells/well, and treated with vorinostat (2.5, 5, and 6.3 μM) which had been dissolved in DMSO, DMEM, or loaded in MCM-41-NH_2_ nanoparticles, for 24 or 72 h. In all cases the effects of vorinostat-loaded nanoparticles were compared to equivalent concentrations of empty nanoparticles. Following treatment, both attached and floating cells were harvested by scraping, pelleted, washed in cold PBS, and the DNA stained by incubation with 50 μg/mL Propidium iodide (PI) in 0.1% sodium citrate, and 0.1% Triton X-100, overnight at 4 °C. The next day, PI-stained cells were analysed by Fluorescence Activated Cell Sorting (FACS) using a FACS Canto II flow cytometer (BD Biosciences, San Jose, CA, USA) equipped with a high throughput sampler (HTS). A total of 10,000 events were recorded per sample, and viable cells were gated from debris using forward and side scatter parameters. The percentage of apoptotic cells was computed using the FLOWJO software (Ashland, OR, USA) by calculating the percentage of cells with a sub-diploid DNA content.

## 3. Results

### 3.1. Characterisation of Nanoparticles

**Figure 1:**
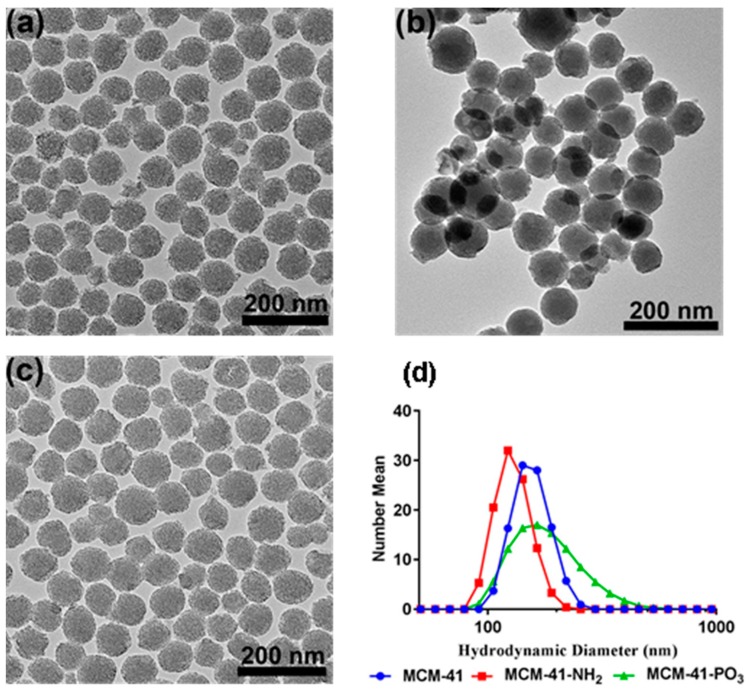
Transmission electron microscopy images of (**a**) MCM-41, (**b**) MCM-41-NH_2_, and (**c**) MCM-41-PO_3_, (**d**) particle size distribution of all three functionalised particles.

The synthesised particles were first characterised using transmission electron microscopy (TEM) to assess the success of the synthesis. As shown in Figure 1, the MCM-41 particles were mostly spherical in shape with a slightly rough outer surface, and hexagonal pore arrangement. Examination of the functionalised amino (MCM-41-NH_2_) and phosphonate derivative (MCM-41-PO_3_) also revealed similar structures with ordered pores, with these modifications having no pronounced effect on the shape or structure of the pores (Figure 1b,c). Furthermore, Figure 1d and Table 1 show dynamic light scattering data in water including intensity mean, number mean, and surface potential of these particles before and after vorinostat loading. All three particles had a mean particle size in the range ~140–200 nm, with the MCM-41-PO_3_ particle (approx. 200 nm) having the largest mean particle size. The MCM-41 and MCM-41-PO_3_ particles had negative zeta potentials of −18 and −40 mV respectively, while the MCM-41-NH_2_ particles had a positive zeta of +20 mV, confirming successful amino functionalisation. Functionalisation of PO_3_ onto MCM-41 significantly reduced the polydispersity index (PDI) to 0.07 compared to MCM-41 and MCM-41-NH_2_ (0.42 and 0.21 respectively). Moreover, even after drug loading, the particle size and zeta potential of all three formulations were largely unaffected except for MCM-41-PO_3_ where encapsulation of vorinostat increased the PDI from 0.07 to 0.2 (Table 1).

**Figure 2:**
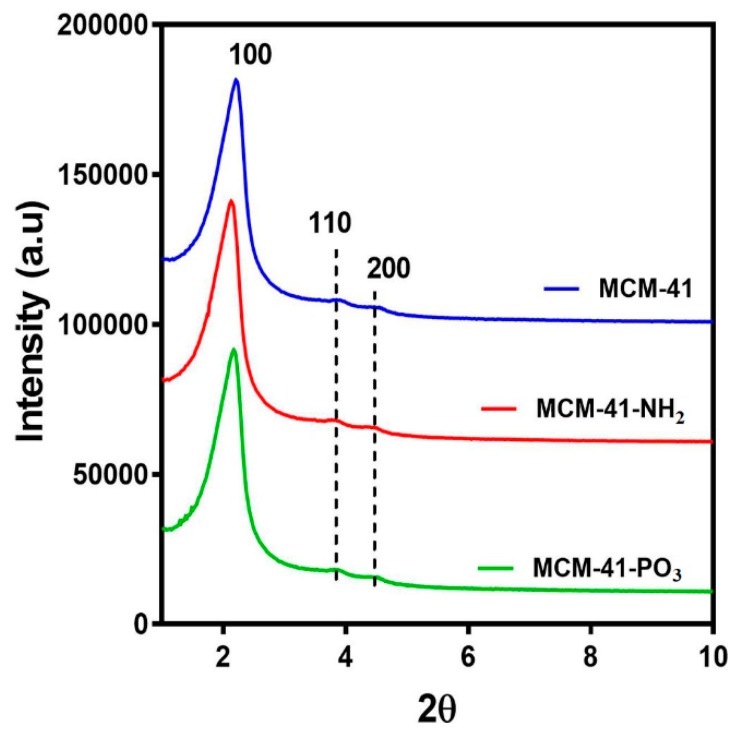
Small angle X-ray Diffraction patterns of MCM-41, MCM-41-NH_2_ and MCM-41-PO_3._

**Figure 3:**
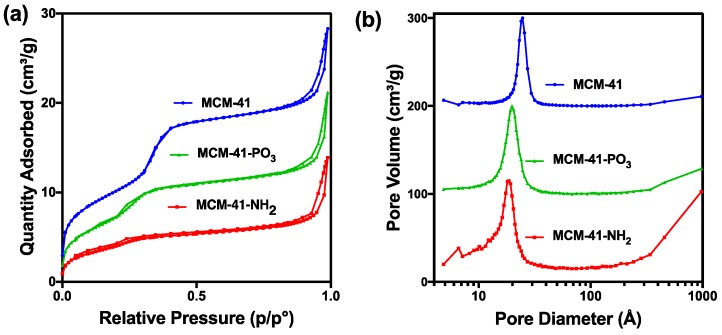
(**a**) Nitrogen sorption analysis of MCM-41 and functionalised particles. (**b**) Pore size distribution of functionalised particles.

X-ray diffractometry (XRD) analysis of the MCM-41 particles and the functionalised NH_2_ and PO_3_ derivatives revealed three well resolved diffraction peaks with a d-spacing ratio close to 1:√3:2. These could be indexed as 100, 110 and 200, confirming the two-dimensional (2D) hexagonal mesopores with a p6 mm symmetry (Figure 2). The position of the diffraction peaks of the NH_2_ and PO_3_ functionalised particles was similar to the pristine particles, although the slight shift of the peaks to the left suggests a small change in the pore size but not in the symmetry [42]. We next performed Nitrogen (N_2_) sorption isotherm analysis, pore size distribution analysis, and BET surface area plots on MCM-41 and their functionalised derivatives. N_2_ adsorption-desorption analysis demonstrated that all particles displayed typical IUPAC type-IV isotherms, indicating the mesoporous nature of the silica samples. The particles also displayed a steep capillary condensation step at a relative pressure (P/Po) range of 0.2–0.4, characteristic of MCM-41 type mesoporous materials (Figure 3a–c). BET surface area plots demonstrated that the pristine MCM-41 had a surface area of 827.9 m^2^/g which was reduced upon functionalisation with APTES (MCM-41-NH_2_) and THMP (MCM-41-PO_3_) to 347.2 m^2^/g and 712.1 m^2^/g respectively (Figure 3a). Similarly, the pore size of MCM-41 (2.4 nm) was reduced to 1.5 nm (MCM-41-NH_2_) and 2.0 nm (MCM-41-PO_3_) following functionalisation (Figure 3b). Finally, pore volume of MCM-41 (0.99 cm^3^/g) was reduced to 0.48 cm^3^/g for MCM-41-NH_2_ and 0.74 cm^3^/g for MCM-41-PO_3_. These changes in physical properties induced by functionalisation of the nanoparticles are consistent with previous reports [43,44]. 

### 3.2. Drug Solubility and Permeability

**Figure 4:**
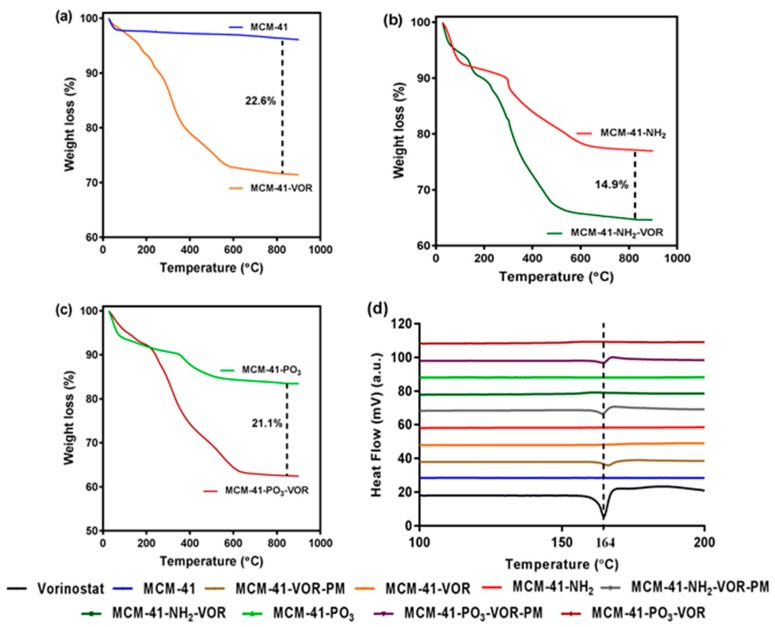
TGA graphs of (**a**) MCM-41 with MCM-41-VOR, (**b**) MCM-41-NH_2_ with MCM-41-NH_2_-VOR and (**c**) MCM-41-PO_3_ with MCM-41-PO_3_-VOR, (**d**) DSC profile of vorinostat, encapsulated vorinostat and physically mixed vorinostat within functionalised particles.

**Figure 5:**
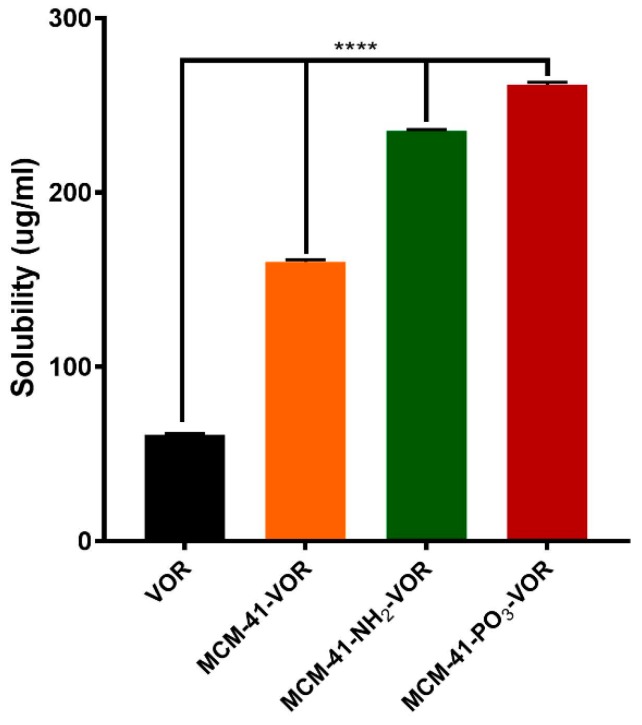
Solubility of vorinostat encapsulated MSNs in water (n = 3 ± SD). **** *p* < 0.0001, ANOVA and Tukey’s post hoc test.

MCM-41, MCM-41-NH_2_ and MCM-41-PO_3_ were then loaded with vorinostat and their theoretical loading capacity of 20% *w*/*w* evaluated using thermogravimetric analysis (TGA) by calculating the weight loss (Figure 4a–c). The loading capacity of vorinostat was 22.6%, 14.9% and 21.1% in MCM-41-VOR, MCM-41-NH_2_-VOR and MCM-41-PO_3_-VOR particles respectively, indicating efficient drug loading in all cases. Once drug loading was established, the solubility of MCM-41-VOR, MCM-41-NH_2_-VOR and MCM-41-PO_3_-VOR was determined by allowing the particles to reach equilibrium in water. The solubility of vorinostat alone in water was 61.06 ± 0.65 µg/mL, which improved by 2.6-fold, 3.9-fold and 4.3-fold when encapsulated in MCM-41-VOR, MCM-41-NH_2_-VOR and MCM-41-PO_3_-VOR particles, respectively (Figure 5). The DSC profile of vorinostat produced a sharp endothermic peak at its melting point confirming its crystalline nature. Similar peaks where observed when vorinostat was physically mixed with nanoparticles at its loading ratio while no such crystalline peaks where noticed for MCM-41-VOR, MCM-41-NH_2_-VOR and MCM-41-PO_3_-VOR particles (Figure 4d), confirming the amorphous nature of vorinostat and further corroborating solubility results.

**Figure 6:**
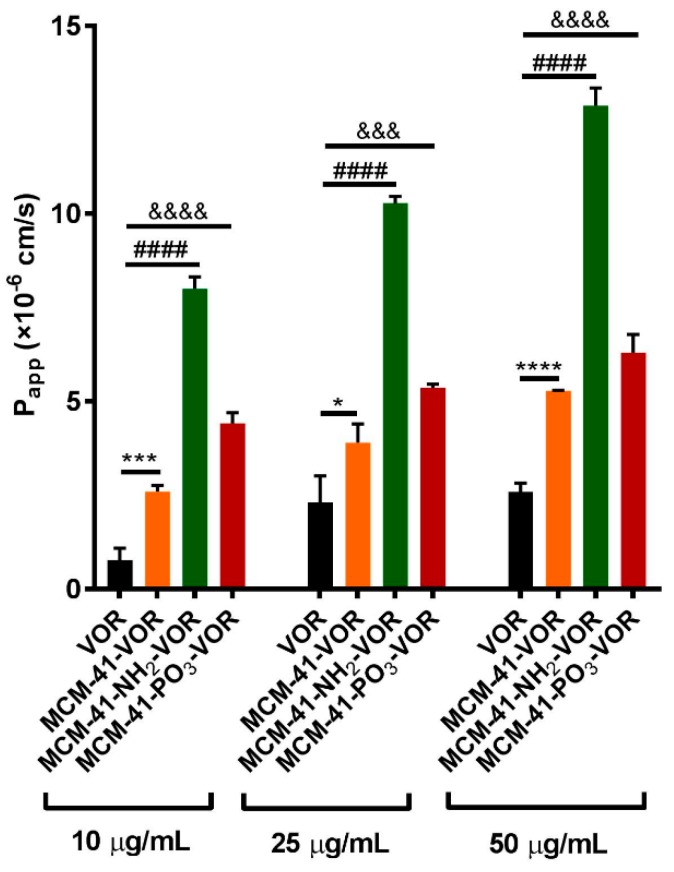
Permeability studies across the Caco-2 monolayer of vorinostat and vorinostat loaded MSNs (n = 3 ± SD) at different concentrations. **** *p* < 0.0001, #### *p* < 0.0001, &&&& *p* < 0.0001, &&& *p* <0.001, * *p* < 0.04, one way ANOVA and Tukey’s post hoc test.

As the permeability of vorinostat is also a major contributing factor which limits its oral application [37], we determined the permeability efficiency of vorinostat alone or vorinostat encapsulated within MCM-41-VOR, MCM-41-NH_2_-VOR and MCM-41-PO_3_-VOR particles in the Caco-2 cell monolayer model. Consistent with our solubility data, encapsulation within all three type of silica resulted in significantly higher P_app_ values compared to free drug (Figure 6). Furthermore, the analysis revealed that vorinostat encapsulated within amino-modified particles displayed the highest permeability (~4-fold) at the highest concentration (50 µg/mL), compared with pre-dissolved vorinostat. To assess the integrity of our monolayer we performed a recovery experiment where after 4 h treatment all particles were removed, the monolayer was washed and replaced with fresh media, and TEER measured over 24 h. As shown in Figure S1, TEER was fully recovered in all samples by 12 h suggesting that the decrease in TEER was transient and reversible.

### 3.3. Assessment of the Anti-Tumour Activity of Vorinostat Encapsulated within Nanoparticles

Having determined that vorinostat encapsulated within MCM-41-NH_2_ nanoparticles (MCM-41-NH_2_-VOR) have the highest permeability and a 3.9-fold increase in solubility, we next compared the HDAC inhibitory activity, effect on gene expression and apoptotic activity of vorinostat encapsulated within MCMI-41-NH_2_ particles, with vorinostat dissolved in media (DMEM) or DMSO. Empty MCM-41-NH_2_ particles served as an additional control.

**Figure 7:**
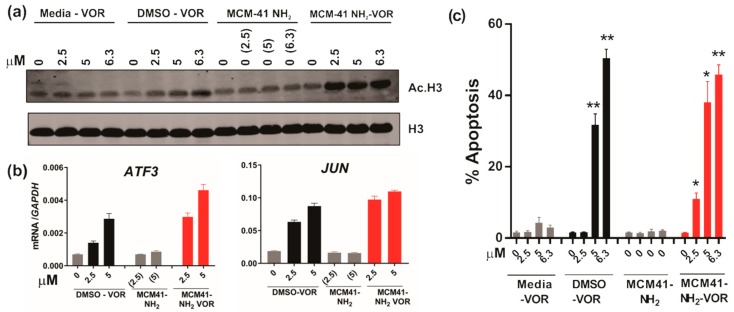
HDAC inhibition and anti-tumour activity of vorinostat dissolved in media, DMSO or encapsulated within nanoparticles. (**a**) Western blot of acetylated histone H3 and total histone H3 following treatment of HCT116 CRC cells with escalating doses of vorinostat dissolved in media, DMSO, or encapsulated within nanoparticles (MCM-41-NH_2_-VOR) for 1 h. Cells treated with corresponding amounts of empty nanoparticles (MCM-41-NH_2_) served as controls. (**b**) mRNA expression of *ATF3* and *JUN* determined by qRT-PCR following 24 h treatment with escalating doses of vorinostat dissolved in DMSO or encapsulated within nanoparticles (MCM-41-NH_2_-VOR). (**c**) Apoptosis induction of HCT116 cells following 24 h treatment with escalating doses of vorinostat dissolved in media, DMSO, or encapsulated within nanoparticles (MCM-41-NH_2_-VOR) was determined by propidium-iodide staining and FACS analysis. Cells treated with corresponding amounts of empty nanoparticles (MCM-41-NH_2_) served as controls. Values shown are mean ± SEM of a representative experiment performed in triplicate. *, *p* < 0.05, **, *p* < 0.01 and ***, *p* < 0.001, unpaired Student’s *t*-test.

Treatment of HCT116 colorectal cancer cells with MCMI-41-NH_2_-VOR resulted in significantly greater induction of histone H3 acetylation compared to vorinostat dissolved in media (DMEM), and was similar to the effect induced by equimolar concentrations of vorinostat dissolved in DMSO. As expected, treatment with empty MCM-41-NH_2_ particles had no effect on histone H3 acetylation (Figure 7a). Having demonstrated that MCM-41-NH_2_-VOR inhibited HDAC activity to a similar extent as vorinostat dissolved in DMSO, we next compared their ability to induce mRNA expression of the established HDACi target genes, *ATF3* and *JUN* in HCT116 cells. Consistent with the effects on histone H3 acetylation, MCM-41-NH_2_-VOR induced *ATF3* and *JUN* mRNA to a similar extent as equimolar concentrations of vorinostat dissolved in DMSO (Figure 7b). 

We next determined the effects of MCM-41-NH_2_-VOR on apoptosis in HCT116 cells. MCM-41-NH_2_-VOR induced a dose-dependent increase in apoptotic cells after 24 h treatment, which was significantly greater than apoptosis induction by equimolar concentrations of vorinostat dissolved in media, and similar to eqimolar concentrations of vorinostat dissolved in DMSO. As expected, treatment with nanoparticles alone had minimal effect on apoptosis (Figure 7c).

**Figure 8:**
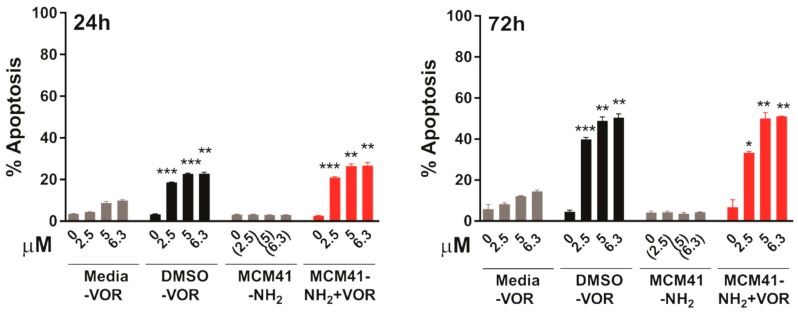
Apoptosis induction in HH cells after 24 h (**left**) or 72 h (**right**) treatment with escalating concentrations of vorinostat dissolved in media, DMSO, or encapsulated within nanoparticles (MCM-41-NH_2_-VOR), determined by propidium-iodide staining and FACS analysis. Cells treated with corresponding amounts of empty nanoparticles (MCM-41-NH_2_) served as controls. Values shown are mean ± SEM of a representative experiment performed in triplicate. *, *p* < 0.05, **, *p* < 0.01 and ***, *p* < 0.001, unpaired Student’s t-test.

Finally, we further validated these findings in the cutaneous T cell lymphoma (CTCL) cell line HH, as Vorinostat is FDA approved for the treatment of CTCL. Similar to the effects observed in HCT116, MCM-41-NH_2_-VOR induced a robust dose-dependent induction of apoptosis after 24 and 72 h, which was significantly higher than equimolar concentrations of vorinstat dissolved in media (Figure 8). Furthermore, MCM-41-NH_2_-VOR induced apoptosis to a similar extent as equimolar concentrations of vorinostat dissolved in DMSO. Collectively, these findings demonstrate that vorinostat encapsulated in MCM-41-NH_2_ nanoparticles induces significantly greater anti-tumour activity compared to vorinostat dissolved in media, and equivalent biological activity to vorinostat dissolved in DMSO.

## 4. Discussion

Poor solubility of small molecules is a major impediment to successful drug development, and the development of efficient drug formulations [1,2]. Nanoencapsulation of poorly soluble drugs has been increasingly explored as a means to improve the physicochemical properties of hydrophobic molecules, thereby improving drug bioavailability. However, traditional nanocarriers suffer from drawbacks such as poor loading efficiency, poor oral stability, burst release and limitation in scalability [2,24,45]. More recently, mesoporous materials have emerged as a new biocompatible drug delivery carriers with the potential to encapsulate a wide variety of molecules for efficient oral drug delivery [7,14,25,29]. 

The HDAC inhibitor vorinostat is approved for the treatment of cutaneous T cell lymphoma, and is currently being trialled in combination with other agents, including immune checkpoint inhibitors, for the treatment of several other cancers [46]. However, limitations of its use include its poor solubility and permeability, and vorinostat currently has to be taken up to 4 times a day when administered orally. Better strategies to enhance the bioavailability of vorinostat are therefore needed. While previous studies have investigated the use of polymeric and lipidic nanoparticles to deliver vorinostat for tumour therapy [38,47,48,49,50], the majority of these studies focused on the intracellular delivery of vorinostat in cancer cells. Recently, Kim et al., encapsulated vorinostat within solid lipid and liquid lipid, and showed marked improvement in oral bioavailability. However, their formulation suffered from low encapsulation capacity (~2–3% loading), and the effect of encapsulation on solubility, permeability and anti-cancer activity was not studied [38,50]. 

In the current study, we demonstrate that MSNs are able to encapsulate large quantities of vorinostat and markedly improve its solubility and permeability, which could be further enhanced using functionalised MSNs with NH_2_ or PO_3_ groups. TEM, N_2_ sorption and XRD analyses demonstrated the particles are highly ordered and that surface functionalisation had little impact on ordered pores, but as expected led to a decrease in surface area and pore volume. DLS data further demonstrated that even after loading 15–20% of drug, particle size was not significantly changed. On the other hand, PDI increased after drug loading, which could be due to the release of some vorinostat during DLS measurement which uses water as dispersant [28]. Furthermore, we demonstrated efficient drug loading of vorinostat into MSNs using a well-established rotatory evaporation method which showed 22.6%, 14.9% and 21.1% for loading into MCM-41-VOR, MCM-41-NH_2_-VOR and MCM-41-PO_3_-VOR particles respectively. The reduced amount of drug loaded in MCM-41-NH_2_-VOR correlated with the decreased surface area and pore volume of these particles [43,44]. A further advantage of mesoporous silica is it allows the drug molecules to stay in confined nanopores in a low-energy state, which improves drug solubility by restricting or slowing drug crystallisation. This is likely due to the high surface area and small pore size of the particles which keeps vorinostat in its amorphous form and increases the solubility as per the Ostwald equation. This was confirmed by comparing the crystalline properties of vorinostat-loaded nanoparticles with a physical mixture of drug/silica at the same weight ratio using DSC, which demonstrated an absence of crystalline peaks of vorinostat at its melting point (~160 °C) in all the nanoparticle loaded formulations. Comparatively, physical mixing of the drug with silica revealed a clear endothermic peak near vorinostat’s melting point, confirming the presence of crystalline drug. We demonstrated that all the nanoparticle-loaded drugs showed significantly higher solubility irrespective of functionalisation, although phosphonate functionalisation produced the highest solubility. One possible explanation for this could be improved interaction of vorinostat’s positive charge groups (pKa = 9.2) with the highly negative PO_3_^−^ groups leading to slower nucleation of particles dispersed in an aqueous environment; however, the interaction of vorinostat with these functional groups requires direct confirmation.

Permeability across the intestinal barrier is another factor which hinders the oral delivery of vorinostat. Several strategies have been employed to improve permeability of small molecules and macromolecules including use of enzyme inhibitors, nanoparticles, microparticles and use of chemical permeation enhancers [51,52,53]. Recently, Juere et al. showed that permeability of poorly soluble BCS class II drug resveratrol was comparable to pre-dissolved drug when it was encapsulated within 90 nm MCM-48 type particles. However, the effect of different functionalised mesoporous particles on permeability of BCS class IV molecules has not been previously investigated [27]. While our findings revealed that encapsulation of vorinostat within NH_2_ functionalised particles increased permeability among the formulations tested, we did not see any effect of MSNs on tight junction function. This suggests that the increased permeability of MSN-loaded vorinostat compared to DMSO-dissolved drug is likely due to other mechanisms such as micropinocytosis or caveolae-mediated transport [54]. In support of this, the uptake of MSNs by Caco-2 cells which is the model system used in this study has been previously demonstrated [55]. Additional studies are needed to determine the specific mechanism by which permeability of MSN-loaded vorinostat is enhanced compared to DMSO-solubilised drug.

Finally, we demonstrate that encapsulation of vorinostat within NH_2_-functionalised nanoparticles robustly induced several of the typical molecular effects of HDACi in two cancer cell line models in vitro, including a CTCL cell line, the disease indication for which vorinostat is clinically approved. This included rapid induction of histone hyperacetylation, indicating effective and specific inhibition of HDACs, and the transcriptional induction of *ATF3* and *JUN* expression, two well-established target genes of HDACi, which have been shown to be induced by HDACi across a range of tumour types. Induction of *ATF3* has also been shown to directly drive HDACi-induced apoptosis, at least in part through transcriptional repression of the pro-survival BCL-XL protein [56]. Finally, consistent with *ATF3* induction, vorinostat encapsulated within nanoparticles induced strong levels of apoptosis in both cell lines.

While the molecular and pro-apoptotic effects of MCM-NH_2_-VOR markedly exceeded the effects of vorinostat dissolved in media (free drug), the magnitude of the effects of MCM-NH_2_-VOR paralleled and sometimes exceed the effects induced by equimolar concentrations of DMSO-dissolved vorinostat. These findings indicate no loss of biological activity when vorinostat is encapsulated within MSN nanoparticles. Importantly, empty nanoparticles alone induced no effect on apoptosis, or on expression of *ATF3* and *JUN*, which can be induced in response to cellular stress, indicating the particles alone are relatively inert. These findings now lay the foundation for future studies to assess the impact of oral delivery of vorinostat encapsulated within MSNs to suppress tumorigenesis in mouse models in vivo compared to free vorinostat. 

## 5. Conclusions

In summary, we demonstrated that encapsulation of the BCS class IV molecule vorinostat within MSNs represents an effective strategy for improving its physicochemical properties. All nanoparticles showed good encapsulation efficiency (15–20%) without affecting the particle size and zeta potential. Functionalising silica with phosphonate group produced the highest solubility (~4.3 fold), while permeability of vorinostat was greatly enhanced by positively charged MCM-41. Furthermore, amino functionalised silica showed improved anti-tumour activity compared to free drug in both colorectal and cutaneous T cell lymphoma cells. Our results demonstrate that functionalised mesoporous silica-based carriers have the potential to improve the oral delivery of BCS class IV drugs.

## Figures and Tables

**Table 1:** Particle size, PDI and zeta potential of vorinostat and loaded vorinostat nanoparticles (n = 3 ± SD).

Sr. No.	Sample	Z Average Size (d.nm)	PDI	Intensity (nm)	Number (nm)	Zeta Potential (mV)
1	MCM-41	334.53 ± 52.15	0.42 ± 0.05	188.6 ± 17.11	165.30 ± 8.05	−17.53 ± 0.44
2	MCM-41-VOR	250.26 ± 16.17	0.38 ± 0.02	146.03 ± 8.77	136.83 ± 7.98	−19.46 ±0.46
3	MCM-41–NH_2_	190.50 ± 8.43	0.32 ± 0.01	154.73 ± 9.69	142.50 ± 8.90	14.33 ± 0.57
4	MCM-41–NH_2_-VOR	174.06 ± 1.94	0.21 ± 0.04	168.13 ± 3.51	150.03 ± 2.04	19.33 ± 0.28
5	MCM-41–PO_4_	238.0 ± 2.98	0.07 ± 0.02	260.80 ± 8.44	201.20 ± 7.76	−40.76 ± 0.40
6	MCM-41–PO_4_-VOR	219.73 ± 1.80	0.20 ± 0.04	214.56 ± 10.06	184.16 ± 3.01	−37.36 ± 0.53

## Supplementary Materials

The following are available online at http://www.mdpi.com/1999-4923/10/4/283/s1, Figure S1: Effect of vorinostat and vorinostat loaded nanoparticles on recovery of TEER values of Caco-2 monolayer over 24h (n = 3 ± SD).
