# Supplementary material for: Enhanced Solubility, Permeability and Anticancer Activity of Vorinostat Using Tailored Mesoporous Silica Nanoparticles
Source: Pharmaceutics. 2018 Dec 17;10(4):283. doi: 10.3390/pharmaceutics10040283 (PMC6321298; doi:10.3390/pharmaceutics10040283)
Supplement: Supplementary file 1 [file pharmaceutics-10-00283-s001.pdf]

pharmaceutics

Enhanced Solubility, Permeability and Anticancer Activity of Vorinostat Using Tailored Mesoporous Silica Nanoparticles

Anand Kumar Meka, Laura J. Jenkins, Mercedes Dàvalos-Salas, Naisarg Pujara, Kuan Yau Wong, Tushar Kumeria, John M. Mariadason and Amirali Popat

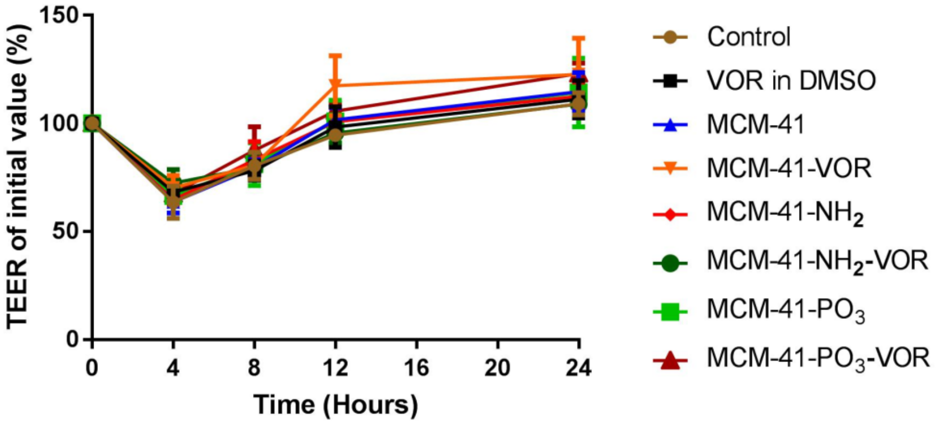

Figure S1. Effect of vorinostat and vorinostat loaded nanoparticles on recovery of TEER values of Caco-2 monolayer over 24h (n = 3 ± SD).
